# Translations, Books, Pamphlets, Etc.

**Published:** 1880-01

**Authors:** 


TRANSLATIONS from the French and German journals will be regularly made, and appear in each issue of this journal, as soon as our exchanges come to hand in sufficient number to furnish the variety of information we desire to present to our readers. Accomplished professional friends have assured us that nothing shall be wanting in this respect, in future issues.

BOOKS, PAMPHLETS, INSTRUMENTS, etc., except dental, intended for notice or review, should be addressed to the senior editor, 147 Edmondson Ave., Baltimore, and those appertaining to dental science should be forwarded to the junior editor, 68 N. Charles St., Baltimore.

MORTUARY STATISTICS, and cognate matters, will receive proper and regular attention in the future.

ARRANGEMENTS are being made to secure summaries of the proceedings of medical and dental societies and associations, which will be published from time to time for the benefit of our readers.

ADVERTISEMENTS, as they appear in this journal, may be relied upon implicitly, as none but those of known merit will be admitted. While the journal is independent in all matters, as set forth in its prospectus, it cannot lend its endorsement to such worthless or unreliable articles and apparatus as sometimes seek publicity through advertising media. We, therefore, invite the attention of our readers to our advertisment department, with full confidence that they will be satisfied with what they may purchase from its notices.

BUFFALO LITHIA WATER.—A most valuable remedy in scarlatina, and the troublesome conditions incident to and dependent upon pregnancy, will be found in this now widely-famous water, and particularly in the water from Spring No. 2. We have been accustomed to prescribe it to the exclusion of *all* other water, from the commencement to the termination of an attack of scarlatina, and have been so much pleased with its action as to esteem it an important factor in the successful management of that much dreaded disease. Its beneficial action in pregnancy is marked and decided, and when females have once experienced its comforting action during gestation, they are sure to resort to it in subsequent pregnancies. Notice of this celebrated water will be found in the advertising department of this journal.

SUMMER RESORTS—NATURAL WATERS.—We will from time to time call attention to the principal summer resorts, their accommodations and the medicinal properties of the most important springs in this country.

